# Surveying Multidisciplinary Aspects in Real-Time Distributed Coding for Wireless Sensor Networks

**DOI:** 10.3390/s150202737

**Published:** 2015-01-27

**Authors:** Carlo Braccini, Franco Davoli, Mario Marchese, Maurizio Mongelli

**Affiliations:** 1 Department of Electrical, Electronic and Telecommunications Engineering, and Naval Architecture (DITEN), University of Genoa, Via Opera Pia 13, 16145 Genoa, Italy; E-Mails: carlo.braccini@unige.it (C.B.); mario.marchese@unige.it (M.M.); 2 National Inter-University Consortium for Telecommunications (CNIT)–University of Genoa Research Unit, Via Opera Pia 13, 16145 Genoa, Italy; 3 Institute of Electronics, Computer and Telecommunication Engineering, National Research Council of Italy (IEIIT-CNR), Genoa Site, Area della Ricerca, Via De Marini, 6-16149 Genoa, Italy; E-Mail: maurizio.mongelli@ieiit.cnr.it

**Keywords:** single-hop wireless sensor networks, distributed coding, decision theory, signal processing, optimization

## Abstract

Wireless Sensor Networks (WSNs), where a multiplicity of sensors observe a physical phenomenon and transmit their measurements to one or more sinks, pertain to the class of multi-terminal source and channel coding problems of Information Theory. In this category, “real-time” coding is often encountered for WSNs, referring to the problem of finding the minimum distortion (according to a given measure), under transmission power constraints, attainable by encoding and decoding functions, with stringent limits on delay and complexity. On the other hand, the Decision Theory approach seeks to determine the optimal coding/decoding strategies or some of their structural properties. Since encoder(s) and decoder(s) possess different information, though sharing a common goal, the setting here is that of Team Decision Theory. A more pragmatic vision rooted in Signal Processing consists of fixing the form of the coding strategies (e.g., to linear functions) and, consequently, finding the corresponding optimal decoding strategies and the achievable distortion, generally by applying parametric optimization techniques. All approaches have a long history of past investigations and recent results. The goal of the present paper is to provide the taxonomy of the various formulations, a survey of the vast related literature, examples from the authors' own research, and some highlights on the inter-play of the different theories.

## 1. Introduction

Multi-terminal source-channel coding in Wireless Sensor Networks (WSNs) arises whenever a number of sensors observe a physical phenomenon represented by one or more random variables, and transmit their measurements over noisy channels to one (or more) sink node(s). The goal of encoding and decoding functions that transform the information emitted by the sources into symbols suitable for transmission over the channels and reconstruct it at the receiving end is twofold: (i) to reduce the possible redundancy intrinsic in the original variables (*source coding*); (ii) to adapt the variables to the channel conditions (*channel coding*). Such operations should be performed in order to minimize the distortion (according to some given measure) between the original and the reconstructed information, under a constraint on the power available for transmission. Information theory aims at finding the fundamental limits attainable by this process, disregarding the possible delay introduced by the encoding-decoding operations and their complexity. Such limits can be attained—at least in the single source-channel case—under Shannon's Separation Theorem [1], by separately performing source and channel coding on the digital representation of the sources.

Still in information theoretic terms, zero-delay (also referred to as “real-time”, “single-letter”, or “instantaneous”) coding is the problem of finding the minimum distortion (according to a given measure), subject to a power constraint, attainable by encoding and decoding functions, with precise limits on delay and complexity. In other words, “the encoder maps every source output symbol separately onto a channel input symbol, and the decoder maps every channel output symbol separately onto a source reconstruction symbol” [2]. In some cases (more specifically, under the conditions stated in [2]), zero-delay coding can indeed be optimal in an unconstrained sense, *i.e.*, even in the class of functions that allow infinite delay and complexity.

The information theoretic approach, though not aiming at finding the optimal coding/decoding strategies, but rather the optimum attainable performance values (minimum average distortion achievable under a given power constraint, or minimum average power to achieve a given distortion), sometimes surprisingly yields an answer to the existence of globally optimal *linear* solutions (*i.e.*, where encoders and decoders are constituted by linear transformations of the observed information). This has been long known for the scalar case of a single Gaussian channel and a single Gaussian source, where the optimum encoder-decoder pair is instantaneous and linear [3]; in [4], Wyner provides a beautiful outline of the reason why this turns out to be so, by equating the rate distortion function *R_eq_*(β) for a certain distortion β to the channel capacity *C_eq_*(α) for a certain average power α. However, once entered the realm of multi-terminal and multi-channel information theory (referred to as *network* information theory), this simple linear (and analog) *joint* source-channel coding (also termed “uncoded” solution, or “Amplify and Forward”—AF), as opposed to the asymptotically optimal source-channel separation in digital communications of [1], occurs only in some special situations. This aspect was thoroughly investigated by Gastpar, Gastpar and Vetterli, *et al.* [5–10], among others.

The recent widespread diffusion of sensor networks and the evolution toward the Internet of Things (IoT) has given new momentum to the investigation of zero-delay distributed source-channel coding (owing to the limited processing and power capabilities of the sensors), and motivates the present paper. In particular, we are interested here in the Physical Layer of WSNs, disregarding other aspects as routing, considerations of node proximity, system organization, latencies and possible losses introduced by the traversal of queues in relay nodes. To keep the discussion more focused we also limit our consideration to the so-called single-hop cases, where direct communication between sensor nodes and a sink is attained, without the presence of relay nodes that characterizes multi-hop WSNs (though similar considerations can be extended to the presence of relay nodes, as well; see, e.g., [11–13]). The term “network”, therefore, is related here to the presence of multiple distributed terminals (the sensors), communicating with a sink over multiple channels (as opposed to the single source-channel case), where the transmitted pieces of information can combine in different fashions, owing to possible interference.

The approach of information theory tries to determine the achievable optimum, without necessarily looking for the coding/decoding strategies yielding it. From another perspective, however, the ultimate goal of the category of problems considered above would be to find the optimal encoder(s)-decoder(s) pairs that minimize the given distortion function under a certain power constraint. The decision theory approach seeks to determine such coding/decoding strategies. Since the decisional agents (encoder(s) and decoder(s)) or “Decision Makers” (DMs) possess different information, though sharing a common goal, the most natural framework here is the functional optimization of team theory [14–16]. Even though the original team problem is *dynamic* [15], in the sense that the encoders' decisions influence the information of the other DMs (the decoders), if the distortion function is quadratic the team can be reduced to a *static* one (*i.e.*, where the decision strategies to be derived by each DM do not influence the information of the others), by keeping into account that the decoder will always compute a conditional mean and by expressing the latter as a functional of the encoders' decision strategies (as will be briefly sketched in Section 5). Nonetheless, the ensuing functional optimization problem still remains formidable. Some insights can be gained by transforming it into a parametric optimization, by means of nonlinear approximating functions (e.g., neural networks [17]).

Between these two visions, a more pragmatic approach consists of fixing the form of the coding strategies and, consequently, finding the decoding strategies and the achievable distortion. In this respect, interesting recent work concerning the application of instantaneous nonlinear mappings at the encoders (not necessarily stemming from a functional optimization problem) regards Shannon-Kotel'nikov (SK) mappings [18]. In the Gaussian Sensor Network (GSN) case, where all random variables (source symbols, measurement and channel noises) have Gaussian distributions, this SK joint analog source-channel coding has been shown to perform better than the linear “uncoded” solution in some cases [19]. Linear solutions optimized in their parameters have been extensively investigated, especially in the Signal Processing literature [20–27], under both GSN and non-Gaussian hypotheses. It is worth noting that, once the coding strategies have been fixed to a linear form, giving rise to a linear conditional mean at the decoder(s), finding the encoders' coefficients that minimize a quadratic distortion function under a power constraint is not a trivial problem. In fact, owing to the presence of such coefficients inside the gain matrices of the decoder(s), the optimization problem turns out to be non-quadratic and, in general, even non-convex.

Summing up, WSNs in which analog source symbols (stemming from measurements of a certain physical phenomenon) need to be collected and transmitted to remote sink stations, are a significant example of systems where network information theory, team decision theory, and distributed estimation can be applied to study different aspects of a multi-faceted problem. All approaches have a long history of past investigations and recent results, the problem has undergone a huge number of formulations and possible variants, all of a certain relevance, and sometimes even slight variations can make the difference between finding a feasible optimal solution or encountering formidable difficulties. The goal of the paper is not to introduce new results, but rather to: (i) provide the taxonomy of the various formulations; (ii) highlight the relevance of analog joint source-channel coding to the field of WSNs; (iii) conduct a survey of the vast related literature; and (iv) show the different points of view introduced by the information theoretic, decision/control theoretic and signal processing approaches. Many survey papers can be found on WSNs in general. Some address computational intelligence [28], data collection [29], and data aggregation [30], which all have some points in common with the environment considered here. However, to the best of our knowledge and with the exception of [7], none treats the zero-delay joint analog coding-decoding problem under multiple points of view.

In the next Section we provide a discussion on the relevance of such type of problems in the WSN field. Section 3 contains an introduction to the multi-terminal source-channel coding problem in WSNs, where multiple spatially separated sensors collect noisy measurements of a physical phenomenon and send them in single-hop fashion to a common sink node over noisy communication channels. Though multiple sinks may be present, the essence of the problem is well reflected in the multi-sensor-single-sink case (multiple encoders and a single common decoder), and we limit our consideration to it. We define the taxonomy of the different problem variants, and we highlight: (i) the measurement process; (ii) the encoding functions; (iii) the channel models; (iv) the distortion function; (v) the decoder structure. In Section 4 we examine the information theoretic approaches to the problem, and survey some of the relevant results. Section 5 deals with the much less investigated team decision theory approaches. We briefly outline the team decision problem in this context, point out where the main difficulties arise in the functional optimization, and how they might be circumvented by suboptimal strategies. Finally, in Section 6, we recall the optimal (parametric) solution in the case where the form of the encoding strategies is fixed. In particular, we focus on linear functions (the “uncoded” case of information theory) under quadratic distortion (LQ). The signal processing literature contains many examples of this particular situation, both in the presence of Gaussian (LQG) and non-Gaussian random variables. We provide a tutorial survey of the problem formulations and of the parametric optimization solutions. Section 7 briefly describes an example, based on the authors' own work, about the non-linearity of the coding/decoding strategies, which bridges, to some extent, the team theoretical and the signal processing aspects. Section 8 contains the conclusions and a classification of the literature surveyed in the different fields.

## 2. Relevance to WSNs

Though the majority of WSNs adopt digital transmission [31] (commonly used standards are IEEE 802.15.4/ZigBee [32], IEEE 802.15.1/Bluetooth [33], ISA100/WirelessHart [34]), a number of solutions based on analog modulation are emerging ([35–41], among others). Although the communication problem presented here is formulated for analog transmission, the impact on WSN applications may be relevant, because fully digital and fully analog architectures may be intrinsically inefficient for a WSN [42]. In multi-hop WSNs with relaying, analog and digital solutions have been compared in [43]. In general, besides the optimality and scalability properties that it exhibits in some cases, analog zero-delay coding (and, in some cases, processing) appears to be convenient where very low power consumption and computational complexity are required.

We summarize here some applications that may be put in relation with the problem addressed by the paper. A set of applications belongs to the family of analog transmission, in particular, when considering hybrid analog-digital architectures. Another one deals with more complex operations performed by the sink, for example, involving a classification task (e.g., target tracking in video surveillance). For the latter case, the following works are relevant. Reference [44] exploits analog joint source-channel coding to drive power allocation while addressing a classification problem at the sink. It highlights how the joint problem of communication and classification needs more sophisticated analytical and numerical tools, as similarly outlined in this paper. Reference [45] addresses the same objective (classification at the sink), by deriving an optimal trade-off between classification accuracy and energy preservation. Again in the detection field, the authors of [43] compare the efficacy of digital *vs.* analog relaying in a sensor network and show that the superiority of digital relaying actually depends on the signal to noise ratio. In a similar hybrid digital–analog (HDA) context, the acoustic sensor network of [35] shows how HDA systems may supersede purely digital transmission in dependence of the radio channel quality. Another analog case is that of [41], in which an AF strategy is applied, together with cooperative coding for the sake of interference mitigation. An analog scatter-radio WSN is presented in [37–39] for environmental monitoring purposes. Extremely low power consumptions over transmission ranges of tens of meters have been achieved.

Analog processing is also adopted for computation. The analog computation for data fusion in [46,47] has also similarities with the framework proposed here, in particular with respect to approaching the problem through functional optimization techniques. In [48], analog signal processing is integrated in a sensor node to simplify the digital computation tasks, thus increasing energy-efficiency; the considered application is vehicle classification. Reference [49] adopts analog computation of Fourier transform coefficients for lower power consumption.

## 3. Taxonomy of WSN Zero-Delay Coding/Decoding Problems

We are interested in zero-delay coding and decoding functions that minimize a given distortion functional (usually a quadratic one) under given constraints on transmission power (or minimize power under a given distortion constraint). As coding and decoding strategies operate on a single realization of the source random variables, our problem has no dynamics over time. To fix ideas, we introduce the problem in the basic Gaussian case. Whenever we deal with non-Gaussian random variables we will state it explicitly. The basic setting we consider comprises a number of sensors that observe a physical phenomenon, whose output can be represented by Gaussian random variables (r.v.'s). The observations are to be transmitted to a sink over one or more noisy channels with some power constraint, and the task of the sink is to provide an estimation of the original variables under a quadratic distortion criterion.

Let ***x*** ∈ ***R****^n^*, ***x*** ∼ *N*(0, ***Σ****_x_*) be the original unknown vector, which we suppose 0-mean and with covariance matrix ***Σ****_x_*, and let ***y*** ∈ *R^m^* be the vector of variables observed at the sink (whatever the observation channel, the action performed by the transmitters, and the transmission channel). Let:

(1) $$\hat{x}=\gamma_{2}(y)$$

be the estimation performed at the sink. Then, since (letting ‖***x***‖^2^ = ***x****^T^* ***x***):

(2) $$\begin{array}{l} \gamma_{2}^{*}(\cdot )=\begin{array}{c} \arg\min\\ \gamma_{2}(\cdot ) \end{array}E\{{\left\|x-\hat{x}\right\|}^{2}\}=\begin{array}{c} \arg\min\\ \gamma_{2}(\cdot ) \end{array}E\{E\{{\left\|x-\hat{x}\right\|}^{2}\}y\}\}=\\ =\begin{array}{c} \arg\min\\ \hat{x} \end{array}E\{{\left\|x-\hat{x}\right\|}^{2}|y\}=E\{x|y\} \end{array}$$

the estimation is always given by the conditional mean (Actually, by Sherman's Theorem [50], the cost function that yields the conditional mean as optimal estimator can be more general. Sherman's Theorem is as follows: Let ***x*** be a random vector with mean ***μ*** and density *f_x_* (·). Let *L*(***e***), ***e*** = ***x*** − ***x̂***, be a loss function such that *L*(**0**) = 0 and *ρ*(***x***_1_) ≥ *ρ*(***x***_2_) ≥ 0 ⇒ *L*(***x***_1_) ≥ *L*(***x***_2_) ≥ 0, where ρ(·) is a nonnegative and convex distance function (e.g., the Euclidean norm). If *f_x_* (·) is symmetric about ***μ*** and unimodal (*i.e.*, it has only one peak), then ***x̂*** = ***μ*** minimizes *E*[*L*(***e***)]. Applied to our conditional mean problem, Sherman's Theorem is: Let *f_x_*_|_*_y_* (·) be symmetric about its mean *E*{***x***|***y***} and unimodal, and let *L*[***e***(***y***)] be a cost function defined as above; then, ***x̂*** = *E*{***x***|***y***} minimizes *E* {*L*[***e***(***y***)]}).

**Figure 1.**
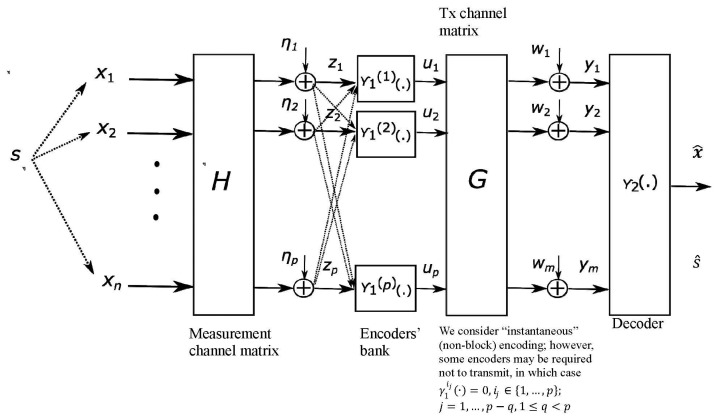
General structure of the WSN zero-delay coding/decoding problem.

In the following, we will introduce the taxonomy of a number of variations of this problem. However, whatever the structure of the problem, if the random vectors ***x*** and ***y*** are Gaussian and the relation between them is linear, then the conditional mean in Equation (2) will be a linear function of ***y*** (or, more generally, if we consider ***x*** to be non-zero-mean, an affine function). We will return on this point in Section 6. We can depict the general configuration of our WSN problem as in Figure 1, along with the basic elements that will be discussed in this classification. With reference to Figure 1, we note the following settings and possible variations of the problem, according to different aspects being considered.

### 3.1. Observation and Measurement Noises

All random vectors ***x***, ***η*** and ***w*** are considered mutually independent.

### 3.2. Original Random Variables and Distortion Functions

There may be an additional “original” variable *s* representing the physical phenomenon, which vector ***x*** may be related to (by mutual correlation, or more generally via a joint probability distribution function; the relation is indicated by dashed lines between *s* and the components of ***x*** in Figure 1). Then, a reconstruction of *s* directly, rather than of the components of ***x***, may be required. It must be noted that in this case the quadratic cost function is the expectation of the square error between two scalar r.v.'s, rather than some quadratic norm of the error between two random vectors. In these cases, the mutual influence between the “source” *s* and the related vector's components that are observed is usually specified in terms of their joint distribution (*i.e.*, in the case of zero-mean jointly Gaussian r.v.'s, by their mutual correlations). There may be, for instance, some distance-dependent correlation function, which characterizes the mutual correlation between the source and each component of ***x*** and between two different components of ***x*** (which may correspond to measurement points spread around the physical phenomenon of interest described by the source *s*). This is the situation considered, among others, in [51–56]. The distortion measures corresponding to the two cases in which the variable of interest is either ***x*** or *s* are, respectively:

(3) $$J_{x}=E\left\{{\left\|x-\hat{x}\right\|}^{2}\right\}$$

(4) $$J_{s}=E\left\{{\left(s-\hat{s}\right)}^{2}\right\}$$

where ‖·‖ is a suitable norm (e.g., Euclidean).

### 3.3. “Expansion” and “Refinement” in Estimation

Two opposite settings are highlighted in [7] with respect to the case of the estimation of ***x***. The first one, called “Expanding Sensor Network” corresponds to the case where the observations (the components of ***z***, or of ***x*** if no observation noise is present) are relatively independent, ***H*** is the identity matrix ***I*** (which also implies *p* = *n*), and matrix ***G*** has a rank of the order of *n*. The term “expanding” derives from the fact that if new sensors were to be added, each new sensor practically brings in new data of interest. In the second situation, called “Refining Sensor Network”, the matrix ***H*** is such that *p* > *n* (in other words, each sensor measures a noisy combination of a relatively small number of variables of interest), so that each sensor adds something to the knowledge of the same group of variables. A case of interest here is that of a relatively “poor” communication infrastructure, with a Multi-Input Multi-Output (MIMO) channel represented by a matrix ***G*** with low rank (with respect to *q*). We will return on these situations in the discussion further on.

### 3.4. Noisy Observations/Multiple Hops

The noisy observation channel may be present or not, mainly to account for measurement uncertainty or “garbled” measurements. It is worth noting that, whereas in the centralized coding case it was shown in [57] (generalizing the earlier result of [58]) that computing the conditional mean of the variables of interest (conditioned to the observation) and using it as the argument of the coding function is optimal, this needs no longer be true in the informationally decentralized coding situation, as noted in [8]. Moreover, in practical applications, the sink might not be the final destination of the information, but rather an intermediate point forwarding the measurements to a processing center. Consider, for example, the case where the sink is a cluster-head collecting measurements from a number of sensors, which should be forwarded to a distant processing center via a satellite link [59]. In these scenarios, involving tandem links, it would be very useful to adopt the definition of link Mean Square Error (MSE) introduced in [60] (as the MSE between the conditional mean estimators of the original signal at the input and output of that link), which allows summing the MSEs of the individual links to obtain the overall MSE.

### 3.5. Power Constraints

The transmission of the encoded variables *u_i_*, *i* = 1, …, *p*, is subject to a power constraint. There are two possibilities.

- An overall power constraint:
  (5) $$E\left\{\sum_{i=1}^{p}u_{i}^{2}\right\}\leq P$$

- Individual power constraints on each transmitted variable:
  (6) $$\begin{array}{cc} E\left\{u_{i}^{2}\right\}\leq P_{i}, & i=1,\ldots ,p \end{array}$$

### 3.6. Zero-Delay Coding

We consider only “instantaneous” (“single-letter”) coding, whereby the coding functions—generally nonlinear—at the sensors are applied to a single measurement individually for a single channel use, rather than to a block of measurements collected over time. The reason for this is essentially to limit the complexity of the code and the ensuing processing burden at the sensors. However, the dashed lines toward the encoders in Figure 1 indicate possible communication among sensors to exchange their measurements, with the arguments of encoding functions $\gamma_{1}^{\left(i\right)}(\cdot ),i=1,\ldots ,p$ , changing accordingly. This yields the possibility to consider different encoding strategies, from completely decentralized (no measurement exchange) to fully centralized ones. The latter case corresponds to the centralized encoding of a Gaussian vector source over a Gaussian vector channel, a problem solved long ago in the linear case (*i.e.*, when the encoder is constrained to be linear) in [61,62]. It is worth noting that the linearly constrained solutions, in the presence of an overall power constraint as in Equation (5), imply that some variables might not be transmitted (from the application of Karush-Kuhn-Tucker optimality conditions to the choice of the optimal coding matrix ***A***), and hence give rise to *q* ≤ *p* encoders.

### 3.7. Transmission Channels' Structure

As already implied by one of the previous points, the structure of the matrix ***G*** (and of the noise covariance matrix ***Σ****_w_*) can model very different types of MIMO channels. ***G*** = ***I*** and diagonal ***Σ****_w_* represent parallel independent channels without interference, also called Orthogonal Multiple Access Channel (MAC) [63]; ***G*** equal to a row vector of all 1's corresponds to the classical MAC (the receiver sees the sum of all channel inputs).

### 3.8. Distortion/Power Minimization

Though still without entering into details, we have outlined so far the situation in which the encoders want to minimize the average distortion under a power constraint. Obviously, the reverse situation is also meaningful to consider, namely, the minimization of average power by using the distortion as a constraint (see, e.g., [24]).

## 4. Information Theory Approaches

As we already noted in the Introduction, Information Theory, though not aiming at finding the optimal strategies, but rather the optimum attainable performance values, sometimes surprisingly yields an answer to the existence of globally optimal linear solutions. In particular, it was shown in [9] that uncoded transmission is strictly optimal in the following case (with reference to Figure 1): a single source, observed *n*-fold (*x_i_* = *s*, ∀*i*), ***H*** = ***I*** (*p* = *n*), $\Sigma_{\eta }=\sigma_{\eta }^{2}I$ , ***G*** = [1, 1,…,1] (*m*=1, *i.e.*, a Multiple Access Channel), distortion as in Equation (4), and constraints as in Equation (6), with *P_i_* = *P*, ∀*i*. The instantaneous linear solution here is not only optimal among single-letter codes, but globally optimal among all arbitrarily long block codes. In this and other situations, uncoded transmission has been proven to scale exponentially better asymptotically with the number of sensors, with respect to digital communication with separate source and channel coding. The result of [9] has been generalized to the asymmetric case of different power constraints and noise, and by considering also the sum power constraint, in [64–66]. Inhomogeneous measurement and transmission channels are considered in [67]. Liu and Ulukus [68,69] determined bounds and an asymptotic scaling law for dense sensor networks transmitting over a cooperative MAC with noise feedback, where, contrarily to the previous cases, separation is order optimal when the underlying random process satisfies some general conditions. In [70], the optimality of uncoded transmission schemes is investigated for two correlated random sources over the Gaussian MAC, whereas [71] characterizes the distortion pairs that are simultaneously achievable on the two source components of a bivariate Gaussian source transmitted to a common receiver by two separate transmitters over an average power-constrained Gaussian MAC, and proves the optimality of uncoded transmission for low signal-to-noise ratio (SNR); the same problem in the presence of perfect causal feedback from the receiver to each transmitter is analyzed in [72]. Rajesh and Sharma consider discrete alphabet sources [73] and the presence of side information over the Gaussian MAC [74]; they also compare three different joint source-channel coding schemes. The same authors study the Orthogonal MAC in [75,76], and the fading Gaussian MAC in [77]. Several types of multiuser channels, not necessarily Gaussian, with correlated source side information are studied in [78], and optimality of separation is proved in those cases for the joint source-channel code, though not yielding the same codes of the source and channel coding problems considered separately.

Recently, Jain *et al.* [79] studied the minimum achievable transmission energy under a distortion constraint. For two correlated Gaussian sources communicated over a Gaussian multiple-access channel, they obtain inner and outer bounds on the energy-distortion region, also showing that uncoded transmission outperforms separation-based schemes in many different conditions. Still in the context of Information Theory, a situation of interest is that of pure source coding, disregarding transmission and channel noise. In a distributed setting like the one we have introduced, this is the framework of the Chief Executive Officer (CEO) problem [80–84].

## 5. The WSN as a Team Decision Problem

The decision theory approach looks for the determination of the strategies, and, since the decisional agents (encoders and decoder) or “Decision Makers” (DMs) possess different information, though sharing a common goal, it must be necessarily in the framework of team theory [14–16,85]. If, to fix ideas, we focus on the minimization of distortion under a power constraint, and we suppose to work with the objective function Equation (3) and constraint Equation (5), the decision problem is:

(7) $$\underset{\gamma_{1}^{\left(1\right)}(\cdot ),\gamma_{1}^{\left(2\right)}(\cdot ),\ldots \gamma_{1}^{\left(p\right)}(\cdot );\gamma_{2}(\cdot )}{\min}E\left\{{\left\|\text{x}-\gamma_{2}(y)\right\|}^{2}\right\}$$

subject to:

(8) $$E\left\{\sum_{i=1}^{p}{\left[\gamma_{1}^{\left(i\right)}(z_{i})\right]}^{2}\right\}\leq P$$

Though this problem falls in the category of dynamic teams [86] (the decisions of the encoders influence the information available to the decoder), it can actually be reduced to a *static* one, by remembering that, indeed, the optimal strategy of the decoder is to compute the conditional mean:

(9) $$\begin{array}{l} \hat{x}=\gamma_{2}(y)=E\left(x(y\right\}=\int_{-\infty }^{+\infty }\xi f_{x(y}(\xi (y)d\xi =\\ =\int_{-\infty }^{+\infty }\xi \frac{f_{y(x}(y(\xi )f_{x}(\xi )}{f_{y}(y)}d\xi =\int_{-\infty }^{+\infty }\xi \frac{f_{x}(\xi )\int_{-\infty }^{+\infty }f_{w}\left[y-G\gamma_{1}\left(H\xi +\zeta \right)\right]f_{\eta }(\zeta )d\zeta }{f_{y}(y)}d\xi \end{array}$$

where we have defined $\gamma_{1}(\cdot )=\text{col}[\gamma_{1}^{\left(1\right)}(\cdot ),\gamma_{1}^{\left(2\right)}(\cdot ),\ldots ,\gamma_{1}^{\left(p\right)}(\cdot )].$

Substitution of Equation (9) into the part to be minimized in Equation (7) yields an expression that is a functional of *γ*_1_(.) only. The reduction to a static team is actually always possible, as was noted long ago by Witsenhausen [87]. Though this fact is sometimes useful and has actually been exploited to specify properties of the solution [88], the still extremely complex form of the expression obtained in our WSN case renders the functional optimization problem formidable, unless some restrictions are imposed on the structure of the encoding functions.

As regards the presence of the stochastic constraint Equation (8), it is worth noting that it can be handled by using Lagrangian duality, in a similar fashion as done in [89] in a different setting. Indeed, one can consider the minimization:

(10) $$\underset{\gamma_{1}^{\left(1\right)}(\cdot ),\gamma_{1}^{\left(2\right)}(\cdot ),\ldots \gamma_{1}^{\left(p\right)}(\cdot );\gamma_{2}(\cdot )}{\min}\left[E\left\{{\left\|x-\gamma_{2}(y)\right\|}^{2}+\lambda \sum_{i=1}^{p}{\left[\gamma_{1}^{\left(i\right)}(z_{i})\right]}^{2}\right\}\right]$$

and then determine the multiplier λ as:

(11) $$\underset{\lambda \geq 0}{\max}\left[E\left\{{\left\|x-\gamma_{2}^{*}(y)\right\|}^{2}+\lambda \sum_{i=1}^{p}{\left[\gamma_{1}^{(i)*}(z_{i})\right]}^{2}\right\}-\lambda P\right]$$

Though posing the problem in a team theory setting is so hard, some simplifications are possible by restricting the form of the strategies. As we have already seen in Section 1, restricting the coding strategies to be linear immediately yields a linear form of the conditional mean at the decoder. As an alternative, it would be interesting to investigate the effect of going the other way round, *i.e.*, forcing the conditional mean to be linear, and trying to find the coding functions that minimize the distortion under the given power constraint in this case. The ensuing static team problem is one with linear information structure, (seemingly) quadratic cost, and Gaussian primitive random variables (LQG). LQG static teams are known to have a globally optimal linear solution [14,85,86]. This can be found by writing the so-called “person-by-person satisfactoriness” (p.b.p.s.) conditions, *i.e.*, the necessary conditions for optimality of the problem in strategic form, and then conditioning expectations to the available information for each agent. For example, in the case of two decisional agents, with common cost to be minimized *J*(*γ*_1_,*γ*_2_), and admissible strategy sets Γ_1_ and Γ_2_, one would write the conditions:

(12) $$\begin{array}{c} E\left\{J\left(\gamma_{1}^{*},\gamma_{2}^{*}\right)\right\}\leq E\left\{J\left(\gamma_{1},\gamma_{2}^{*}\right)\right\},\forall \gamma_{1}\in \Gamma_{1}\\ E\left\{J\left(\gamma_{1}^{*},\gamma_{2}^{*}\right)\right\}\leq E\left\{J\left(\gamma_{1}^{*},\gamma_{2}\right)\right\},\forall \gamma_{2}\in \Gamma_{2} \end{array}$$

which can be transformed into ordinary minimizations by conditioning expectations:

(13) $$\begin{array}{c} \underset{u_{1}}{\min}E\left\{J\left(u_{1},\gamma_{2}^{*}\right)|z_{1}\right\}\\ \underset{u_{2}}{\min}E\left\{J\left(\gamma_{1}^{*},u_{2}\right)|z_{1}\right\} \end{array}$$

In the LQG case (where the observations *z*_1_ and *z*_2_ depend linearly on the primitive r.v.'s), by guessing linear strategies, substituting them in the minimization problems and computing expectations leads to the solution of a linear system (in the unknown parameters that constitute the matrices of the linear strategies); the solution turns out to be unique and hence, given the convexity of the cost and the symmetry of the probability distributions, it is also the globally optimal one.

Now, going back to our case, though it is true that all the assumptions are there, the dependence of the cost on the parameters of the matrices representing the encoders' strategies would be non-quadratic, since these parameters would appear within the expression of the gain matrix at the decoder (matrix ***B*** in Equation (17) of Section 6 below), which is required to compute the linear Minimum Mean Square Error (MMSE) estimator. This (besides the presence of the power constraint) gives rise to a non-classical structure of the static team optimization problem, which would not necessarily imply the existence of globally optimal linear solutions. Forcing also the encoding strategies to be linear (which implies that matrix ***B*** assumes the form of Equation (22) in Section 6), would lead to the same non-convex optimization problem considered and solved in [23].

## 6. Signal Processing and Parametric Optimization

Whatever the structure of the problem, if the random vectors ***x*** and ***y*** are Gaussian and the relation between them is linear, then the conditional mean in Equation (2) will be a linear function of ***y*** (or, more generally, if we consider ***x*** to be non-zero-mean, an affine function):

(14) $$\hat{x}=E\{x|y\}=\gamma_{2}^{*}(y)=By+b$$

In this case, the constant vector ***b*** and matrix ***B*** are easily determined by the condition of having an unbiased estimate and by the orthogonality principle [50], respectively:

(15) $$\begin{array}{l} E\{\hat{x}\}=E\{x\}=\bar{x}\Rightarrow B\bar{y}+b=\bar{x}\Rightarrow b=\bar{x}-B\bar{y}\\ \hat{x}=\bar{x}+B(y-\bar{y}) \end{array}$$

Since Equation (15) shows that we can always work with zero-mean vectors if we consider the shifted variables ***x*** − ***x̄***, ***x̂*** − ***x̄***, ***y*** − ***ȳ***, we will consider zero-mean r.v.'s for the sake of simplicity. The orthogonality principle states that the estimation error is orthogonal to the data:

(16) $$\begin{array}{l} E\{(x-\hat{x})y^{T}\}=0\\ \begin{array}{cc} E\{(x-By)y^{T}\}=0; & BE\{yy^{T}\}=E\{xy^{T}\} \end{array} \end{array}$$

so that, if the covariance matrix of ***y*** is positive definite:

(17) $$B=E\{xy^{T}\}E{\left\{yy^{T}\right\}}^{-1}$$

The MMSE linear estimator defined by Equation (14) (where we now consider ***b*** = 0) and Equation (17) is a discrete Wiener filter.

The calculation of the covariance matrices depends on the (linear) structure of the observation (measurement by the sensors) and transmission (from the sensors to the sink) channels.

Let:

(18) z=Hx+η

represent the measurement process, with ***z*** ∈ ***R****^p^*, ***η*** ∈***R****^p^*, ***η*** ∼*N* (0, ***Σ****_η_*). In general, we can suppose each sensor to observe a noisy linear combination of the variable(s) of interest. Moreover, let:

(19) y=GAz+w

where ***w*** ∈ ***R****^m^*, ***w*** ∼ *N* (0, ***Σ****_w_*) and the matrices *_A_* ∈ *R^qp^* and *_G_* ∈ *R^mq^* represent the linear coding and the effect of the transmission channels, respectively (in general, a linear combination of transmitted variables represents interference). The matrix ***A*** would be diagonal (or block-diagonal, if the individual sensors' observations are allowed to be vectors) if no sensor cooperation (by exchanging measurements) is allowed. Given such structure, and supposing all noise vectors to be mutually independent and independent of ***x***, then:

(20) $$\begin{array}{l} E\{yy^{T}\}=E\left\{\left(GAHx+GA\eta +w)(x^{T}H^{T}A^{T}G^{T}+\eta^{T}A^{T}G^{T}+w^{T}\right)\right\}=\\ =GAH\Sigma_{x}H^{T}A^{T}G^{T}+GA\Sigma_{\eta }A^{T}G^{T}+\Sigma_{w} \end{array}$$

and:

(21) $$E\{xy^{T}\}=E\{x(x^{T}H^{T}A^{T}G^{T}+\eta^{T}A^{T}G^{T}+w^{T})\}=\Sigma_{x}H^{T}A^{T}G^{T}$$

so that:

(22) $$B=\Sigma_{x}H^{T}A^{T}G^{T}{\left(GAH\Sigma_{x}H^{T}A^{T}G^{T}+GA\Sigma_{\eta }A^{T}G^{T}+\Sigma_{w}\right)}^{-1}$$

Sometimes, Equation (22) is written in a different form, which is derived by using the Matrix Inversion Lemma (***WY****^T^* (***X*** + ***YWY****^T^*)^−1^ = (***W***^−1^ + ***Y****^T^****X***^−1^***Y***)^−1^ ***Y****^T^****X***^−1^):

(23) $$B={\left[\Sigma_{x}^{-1}+H^{T}A^{T}G^{T}{\left(GA\Sigma_{\eta }A^{T}G^{T}+\Sigma_{w}\right)}^{-1}GAH\right]}^{-1}H^{T}A^{T}G^{T}{\left(GA\Sigma_{\eta }A^{T}G^{T}+\Sigma_{w}\right)}^{-1}$$

If the source statistics is unknown, the Best Linear Unbiased Estimator (BLUE) can be used instead of the MMSE estimator. In this case, the second expression is readily useful as:

(24) $$B_{BLUE}={\left[H^{T}A^{T}G^{T}{\left(GA\Sigma_{\eta }A^{T}G^{T}+\Sigma_{w}\right)}^{-1}GAH\right]}^{-1}H^{T}A^{T}G^{T}{\left(GA\Sigma_{\eta }A^{T}G^{T}+\Sigma_{w}\right)}^{-1}$$

We further note that, in the linear cases considered in this section, and still assuming 0-mean variables, the estimation error would be given by:

(25) $$\begin{array}{l} E\left\{{\left\|x-\hat{x}\right\|}^{2}\right\}=E\left\{{\left\|x-By\right\|}^{2}\right\}=E\left\{{\left(x-By\right)}^{T}(x-By)\right\}=\text{tr}E\left\{(x-By){\left(x-By\right)}^{T}\right\}=\\ =\text{tr}\left[E\left\{(x-By)x^{T}\right\}-E\left\{(x-By)y^{T}\right\}B^{T}\right]=E\left\{x^{T}(x-By)\right\}=\\ =\text{tr}\Sigma_{x}-\text{tr}E\left\{Byx^{T}\right\}=\text{tr}\Sigma_{x}-\text{tr}E\left\{B\left(GAHx+GA\eta +w\right)x^{T}\right\}=\\ =\text{tr}\Sigma_{x}-\text{tr}BGAH\Sigma_{x} \end{array}$$

The above relations, which are classical ones in linear estimation theory, can be easily modified to the case where the original phenomenon of interest is represented by a single source observed by multiple sensors, which we also considered in Section 3, as depicted in Figure 1.

As a final general note, we recall that the orthogonality principle is related to the quadratic distortion measure, irrespectively of Gaussianity in the underlying r.v.'s; therefore, all relations we have summarized remain valid with respect to *linear* estimation, *i.e.*, when the optimal encoders and decoder are restricted to be linear functions of their observations. In the Gaussian case, the linear optimal decoder coincides with the conditional mean, under linear (AF) encoding functions. However, as we have seen in Section 4, even under Gaussian hypotheses, linear encoders and decoders turn out to be globally optimal only in some special cases. Very recently, necessary and sufficient conditions have been derived for linearity of (centralized) optimal mappings, given a general noise (or source) distribution, and a specified power constraint [90].

In the general setting that we have described so far, much work has been done in the case of linear (AF) coding and linear decentralized estimation (combined optimization of decoder and coders' matrices), in both Gaussian and non-Gaussian cases. Energy optimized AF is considered in [20,21] with the BLUE estimator for a scalar source with *K*-fold observations transmitted over the Orthogonal MAC; [21] also derives the optimal power allocation with the sum power constraint and the minimum power under zero-outage estimation distortion constraint. Here too, as in the centralized cases of [61] and [62], the application of Karush-Kuhn-Tucker conditions in the optimal power allocation with the sum power constraint can lead to completely turn off “bad” sensors (with poor channels and observation quality). Owing to the adoption of the BLUE estimator, the source probability distribution can be unspecified. In a different approach, reference [22] supposes sensors' observations to be quantized, and finds the optimum quantization levels and transmit power levels under an MSE constraint. Reference [23] studies the optimum linear decentralized estimation problem under coherent (sum) and orthogonal MAC, in the cases of scalar and vector observations, under general noise. In the scalar case, they derive the optimum power allocation for the coherent MAC and also compare the result to the orthogonal MAC (interestingly, in the coherent MAC case the optimum solution does not turn off any sensors). In the vector case, the linear optimization problem for the orthogonal MAC was shown to be NP-hard in [25] (we recall that this is due to the non-convexity of the optimization problem derived by substituting the conditional mean—see Equation (9)—in the quadratic error function, even in the linear case). The optimal solution in the absence of channel noise is derived analytically (in closed form) in [23] for the coherent MAC case; under noise, the problem of finding the optimal modulators' matrices is formulated as a Semi-Definite Programming (SDP) one, and the effect of power and bandwidth constraints is investigated. In [26], by adopting similar channel models with power and bandwidth constraints as in [23], the optimum linear modulator matrices are found that minimize the MMSE gap between the system over a noisy channel and the one over a noiseless channel, giving rise to a water-filling solution. Always in the general case, optimum linear estimators are derived in [27]. Ribeiro and Giannakis treat both the Gaussian [91] and the general case of unknown probability densities [92]. A complete network problem, considering different protocol layers, is treated in [93].

Back to the GSN case, [94,95] consider distributed analog linear coding of multiple correlated multivariate Gaussian sources over the coherent MAC. Chaudhary and Vandendorpe [96] address the optimization of AF gain coefficients (*i.e.*, the power allocation) under two different settings: (i) exact Channel State Information (CSI), where the fading attenuation coefficients of the transmission channels are known at the encoders and decoder; (ii) imperfect CSI, where the fading coefficients are estimated. They derive an original algorithm based on the successive approximation of the linear MMSE distortion, which is computationally efficient and exhibits very good convergence properties. In [97], the same authors consider a similar problem, but under quantization of the transmitted variables, rather than analog transmission. Their goal is the design of joint quantization and power allocation to minimize MSE distortion for a given total network power consumption. The 1-bit quantization for decentralized estimation of an unknown parameter in the presence of an orthogonal MAC is treated in [98] for both Gaussian and Cauchy channel noise.

The lifetime maximization is studied in [99] in TDMA and interference limited non-orthogonal multiple access (NOMA) channels as a joint power, rate and time slot (for TDMA) allocation problem under various constraints.

Very interesting recent work concerning the application of instantaneous nonlinear mappings at the encoders (not necessarily stemming from an optimization problem) regards Shannon-Kotel'nikov mappings [18,19,100–103]. The GSN case with joint analog source-channel coding is considered in [19], and it is shown to perform better than the uncoded solution.

## 7. An Example of Non-Linear Coding/Decoding Strategies

An example of non-linear coding/decoding strategies is outlined now, in order to emphasize the complexity of the problem even under a small number of sensors, and to highlight the contribution from the fields of team decision theory and signal processing. The 1:2 bandwidth expanding system of [18] is used to address cost Equation (4) with respect to the source estimation with *p* = 2. Each source measurement *z* is mapped by two sensors on the double Archimedes' spirals ***u*** ∈ *R*^2^ as follows:

(26) $$u(z)=\pm \frac{\Delta }{\pi }\varphi (\alpha z)\left[\cos(\varphi (\alpha z))i+\sin(\varphi (\alpha z))j\right]$$

where *α* is a gain factor, *φ*(·) is the inverse curve length approximation $\varphi (\xi )=\pm \sqrt{\frac{\xi }{0.16\Delta }}$ (subsection III.B of [18]) and Δ is the (radial) distance between the two spiral arms. The outputs of the sensors are the components of vector ***u***, subject to constraint Equation (6). The coding operation consists of a bandwidth expansion, because the source *s* ∈ *R* is mapped into ***u*** ∈ *R*^2^. More specifically, *s* defines the angle of the spiral through the application of *φ*(·) and the Cartesian coordinates of the spiral ***u*** are sent over the channels. The decoding operation at the sink consists of finding (in polar coordinates) the angle corresponding to the point on the spiral closest to the received signal ***y***. The parameters *α* and Δ are free variables defining the shape of the spirals; they are set to minimize distortion, while satisfying the power constraint. Under Gaussian hypotheses, the setting may be driven by closed-form expressions. In more general environments, numerical approximation should be provided. The technique belongs to the already mentioned category of SK mappings.

In order to allow more general nonlinear coding-decoding strategies and compare them with the results of SK, we consider here the approach of [56], which we briefly summarize. Coding and decoding strategies for the estimation of *s* are of the form:

(27) $$\begin{array}{ccc} u_{i}={\hat{f}}_{i}(x_{i},\omega_{i}), & i=1,\ldots ,p; & \hat{s}=\hat{g}(y,\omega_{g}) \end{array}$$

where we choose *f̂_i_*(·) and *ĝ*(·) to be neural networks (NNs) depending on the choice of the basis functions (e.g., sigmoid) of each layer; ***ω****_i_* and ***ω****_g_* are vectors of parameters activating the basis functions [104]. Among the various possible fixed-structure for coding-decoding functions, NNs have been chosen for their powerful approximation capabilities. Vectors ***ω****_i_* and ***ω****_g_* should be numerically optimized in a joint process in virtue of the team structure of the problem [105]. The technique is identified by the acronym NN in the following.

Some additional remarks may be useful to clarify the basic differences between the two nonlinear strategies. The SK coding is centralized, since the projection operation onto the spirals needs the joint knowledge of the two sensors' inputs. The NN coding process may work in two ways. In the centralized approach, each sensor *i* knows the input vector ***x*** of all the sensors. In the decentralized one, it knows only its own input *x_i_* as evidenced in Equation (27). SK defines coding functions in polar coordinates, while NN defines coding functions in Cartesian coordinates. SK has limitations in the number of coding components, *i.e.*, *p* = 2, 3; an insightful discussion on how a further generalization may be tricky is presented in section V of [106]. The NN may be scalable to any *p*; the most severe limitations to scalability reside in the complexity of the minimization process in terms of (offline) computational effort, in the choice of an appropriate setting of the several parameters affecting the numerical process, and in the presence of local minima.

**Figure 2.**
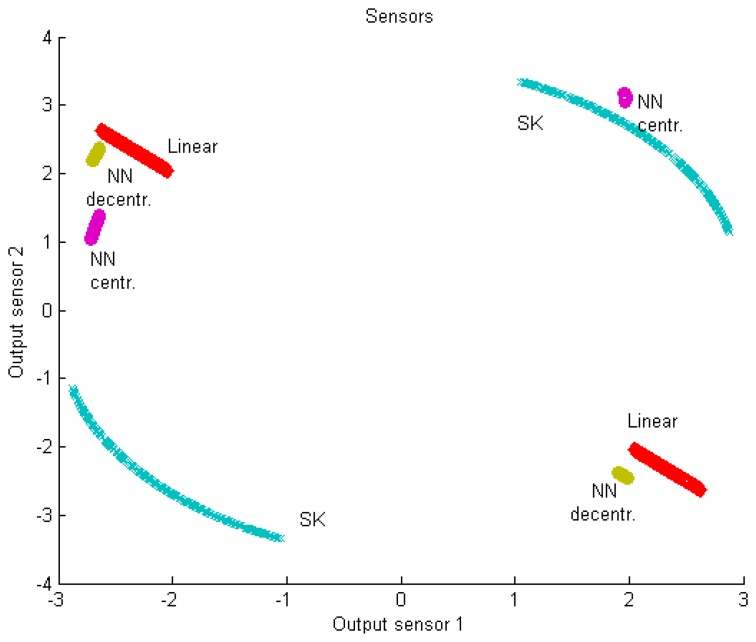
Single source with bimodal distribution: coders.

**Figure 3.**
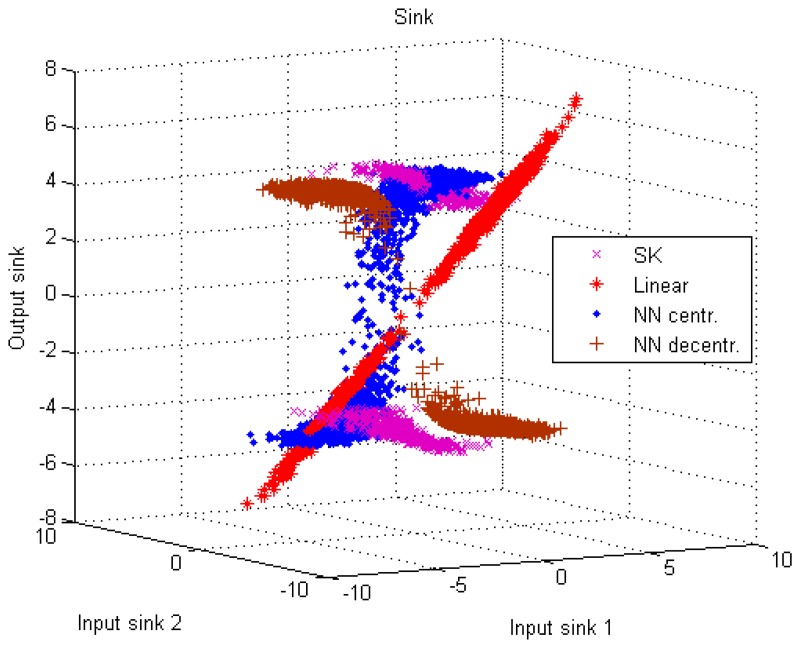
Single source with bimodal distribution: decoder.

All the available strategies (linear, SK, centralized and decentralized NN) have been tested in [105] with respect to a bimodal distribution in *s*. The distribution consists of the superposition (with equal probabilities) of two uniform distributions in [−4.5, −3.5] and [3.5, 4.5]. No measurement noise is considered, channel noises are independent normal distributions with unit variance, channel gains are set to 1. The total available power is 11. In SK, α* =3.31, Δ* =1.32, which have been found numerically. To help SK in the decoding phase, the angle interval (in polar coordinates) over which we search for the spiral point closest to the received point ***y*** has been restricted to the one generated just before the addition of the channel noise. Otherwise, ***y*** is projected back to the wrong spiral arm and the effect on distortion is dramatic. This corresponds to a-priori deleting the *threshold* distortion component of SK (subsection II.C of [18]). As far as the NN is considered, the hyperbolic tangent is used as activation function with 30 hidden neurons in the sensors and 20 hidden neurons in the sink (a single coder with 30 hidden neurons is used in the centralized version). In contrast to our previous tests in reference [105], here we have chosen to also introduce constraints of type Equation (6) in the NN (with the total power divided equally between the two encoders), in order to force the strategy to conform to the bandwidth expansion in all cases. In fact, when this is not done, both NN approaches tend to turn off one sensor and apply a constant amplification factor to the input signal on the other sensor (this is quite evident under the centralized NN in [105]), whereas a larger coding range is generated by SK. Figures 2 and 3 show the coders and decoder, respectively, under the different strategies. The distortion is 1.33 under the linear approach (in [105] we reported a value of 1.247, because we used a slightly higher power allowance in the linear case, to be fair with respect to the NN approaches, where the global power constraint is accounted for by a penalty function that gives rise to a looser enforcement; this is not needed here, as the penalty functions on individual power constraints in the NNs appear to be respected more sharply). The values corresponding to the other strategies are: 0.117 with SK, 0.866 with centralized NN and 0.140 with decentralized NN (the surprisingly higher value for the centralized case is probably due to the enforcement of the additional individual constraints that further reduce the degrees of freedom; the corresponding values obtained in [105] without the additional constraints were 0.133 and 0.155, respectively, but tended to deviate significantly from the bandwidth expansion). The nonlinear curve trend of the SK and NN decoders is quite evident. Despite their different coding behaviors, the nonlinear decoding strategies are quite similar. The further generalization of the results to the presence of measurement noise ***η*** shows a higher robustness of the NN with respect to SK [105].

## 8. Conclusions

Despite the apparent simplicity intrinsic in its formulation, zero-delay distributed coding in WSNs is a problem that opens up a surprisingly large spectrum of approaches and interpretations. Different points of view can be perceived by stating it in the framework of disciplines like Information Theory, Team Decision Theory, or Signal Processing. We have attempted to highlight such different viewpoints and formulations in surveying the literature on the topic, in the light of the taxonomy of problem versions introduced in Section 3. The main approaches found can be summarized as in Table 1 below. Besides the general philosophy pertaining to one or the other of the three disciplines considered, we have classified the papers in the literature with respect to the type of transmission channel setting (coherent or orthogonal MAC) and to the distortion measure (scalar or vector). The latter aspect is meaningful to characterize the interest (with respect to the situation depicted in Figure 1) either in the estimation of the random variable representing the very source of the physical phenomenon, or in the estimation of the multiple spatial realizations that are observed by the sensors. As regards the information theoretic formulations, we have distinguished the cases in which fundamental limits are derived and those where the optimality of the AF solution can be proved. Among the Signal Processing methodologies, we have separated the cases regarding: (i) the search of optimal linear encoders and decoder; (ii) the application of nonlinear parametric optimization methods to approximate optimal nonlinear coding and decoding functions (acting on continuous random variables); (iii) the search of optimal quantized encoders.

The optimality of the linear solution has been proved in some cases of the coherent MAC, but no similar results seem to exist for the case of the orthogonal MAC. Structural results exist for problems that are, in principle, substantially more complex than the setting we have considered here, as they involve system dynamics and feedback control systems. The recent book by Yüksel and Başar [85] contains a bulk of results that admirably blend decentralized (team) control theory and information theory and highlight the role of information structures, as summarized also in [107]. We have not gone into details of these aspects; some of the references in [85] are related to our WSN class of problems, and we have indicated it in Table 1. The richest literature referring to the setting represented in Figure 1 appears to be that of Signal Processing, where mostly parametric optimization methods are applied to fixed-form strategies.

Finally, we remark again the relevance of this problem setting for multi-terminal coding in the Physical Layer of WSNs. Notwithstanding the optimality of the zero-delay coding solutions in some cases (often combined with linearity), the application of such analog joint source-channel coding may result particularly attractive in situations where very low power consumption and computational complexity are required, as often happens in WSN deployments in harsh or hardly accessible environments.

## Figures and Tables

**Table 1.** Classification of different WSN approaches (in the setting studied in this paper) in the literature.

		**Coherent MAC**	**Orthogonal MAC**	**Scalar Distortion Measure**	**Vector Distortion Measure**
Information Theory	Optimal solution	[9,64–67]		[9,64–67]	
	Fundamental limits	[68–74,76–79]	[75]	[68,69]	[70–79]
Decision Theory			[53–56,85,105] and references therein	[85,105] and references therein	[53–56,85,105] and references therein
Signal Processing	Linear encoders (AF)	[23,26,27,94,95]	[20,21,23,25,27,96]	[20,21,96]	[23,25–27,94,95]
	Nonlinear parametric optimization		[18,19,53–56,100–103,105]	[12,18,105]	[18,19,53–56,100,102,105]
	Quantized encoders		[22,91,92,97,98]	[22,91,97,98]	[92]
